# Effect of Pulsed Electric Field on the Electrodialysis Performance of Phosphate-Containing Solutions

**DOI:** 10.3390/membranes12111107

**Published:** 2022-11-05

**Authors:** Olesya Rybalkina, Ksenia Solonchenko, Daria Chuprynina, Natalia Pismenskaya, Victor Nikonenko

**Affiliations:** 1 Physical Chemistry Department, Kuban State University, 149 Stavropolskaya Str., 350040 Krasnodar, Russia; 2 Analytical Chemistry Department, Kuban State University, 149 Stavropolskaya Str., 350040 Krasnodar, Russia

**Keywords:** electrodialysis, pulsed electric field, anion-exchange membrane, phosphates, energy consumption, scaling

## Abstract

A comparative analysis of mass transfer characteristics and energy consumption was carried out for the electrodialysis recovery of P^V^ from of NaH_2_PO_4_ solutions and multicomponent (0.045 M Na_x_H_(3−x)_PO_4_, 0.02 M KCl, 0.045 M KOH, 0.028 M CaCl_2_, and 0.012 M MgCl_2_, pH 6.0 ± 0.1) solution in conventional continuous current (CC) and pulsed electric field (PEF) modes. The advantages of using PEF in comparison with CC mode are shown to increase the current efficiency and reduce energy consumption, as well as reduce scaling on heterogeneous anion-exchange membranes. It has been shown that PEF contributes to the suppression of the “acid dissociation” phenomenon, which is specific for anion-exchange membranes in phosphate-containing solutions. Pulse and pause lapse 0.1 s–0.1 s and duty cycle 1/2 were found to be optimal among the studied PEF parameters.

## 1. Introduction

Electrodialysis (ED) is finding more and more applications as part of hybrid membrane processes providing closed production cycles for water [1,2], ammonium, and phosphates [3,4], as well as proteins, phospholipids, and other valuable nutrients [5,6,7,8]. A significant part of liquids that undergo ED demineralization [9], concentration [10], or pH correction [6,9,11] are multicomponent solutions. As a rule, these solutions contain calcium, magnesium cations, and anions of polybasic organic and/or inorganic acids [11,12], capable of fouling ion-exchange membranes [13,14]. Fouling causes a sharp increase in energy consumption [15], violation of hydrodynamic regimes [16], and leads to replacement of membrane stacks, which reduces the environmental and economic attractiveness of electrodialysis. Metathesis [17,18] or electrodialysis reversal [19,20], as well as antiscalants [21,22] and cleaning reagents [23], are used to suppress scaling (mineral type of fouling). In addition, ion-exchange membranes (IEMs) with special surface characteristics are being developed [21,24], and new current modes are proposed, for example, feed-forward voltage-control [25], when the potential drop decreases in proportion to the solution demineralization, etc. The use of pulsed electric field (PEF) instead of conventional ED, which is carried out under a direct conventional continuous electric current (CC) or a potential drop, seems to be the most promising [26]. The PEF mode, in which electrical pulses of direct continuous current (or potential drop) alternate with a pauses during which the current is zero, was proposed by Karlin and Kropotov [27] in 1995 and aroused great interest lately. Recent studies, summarized in the review [28], give the following mechanism of the influence of the PEF on the electrodialysis performance. During a pause lapse (when the applied current or potential drop is equal to zero), the depleted and enriched diffusion boundary layers (DBLs) in the solution near the IEM surface partially “dissipate”. The near-surface concentrations of electrolytes at the enriched DBL side decrease, that is, go from the “danger zone”, in which a local excess of the solubility product of some salts is achieved, causing their precipitation [19]. The concentrations of electrolytes in the depleted DBL at the IEM surface increase, causing a decrease in water splitting. A local shift in the pH of the solution leads to a reduction of the electrostatic interactions of proteins with the membrane surface [28], as well as the dissolution of some (pH sensitive) foulants [29]. In addition, fluctuations in the surface concentration of electrolytes create electrical inhomogeneity, which contributes to a faster (and more intense) development of electroconvection compared with CC [30]. Electroconvection mixes the depleted solution, providing additional delivery of the target component to the IEM surface as well as preventing scaling and membrane fouling by organic components. Thus, the effect of PEF on ED performance is a combination of three phenomena: the reduction of concentration polarization, the suppression of water splitting, and the promotion of electroconvection [28,29,31,32]. The specific selectivity of the membrane does not depend on the mode (CC or PEF) if the average current density in both cases remains the same [33]. 

The formation and relaxation of concentration profiles at the IEM is a dynamic process. Therefore, the ratio between the duration of an electric field pulse and the pause lapse (duty cycle), as well as the amplitude and pulse lapse, strongly depends on the chemical nature of the substances subjected to ED [9,31,32,34].

Note that many of the liquid media subjected to ED treatment, such as milk whey, bovine blood serum, liquid digestates (the product that remains after anaerobic fermentation of biodegradable raw materials) of municipal wastewater or animal waste products [20,35,36,37], and other residual streams contain anions of polybasic acids, including phosphates. In recent years, it has become known that the presence of these electrolytes in solutions provokes an intense generation of H^+^, OH^−^ ions [38], contributes to the reduction of electroconvection [39] compared with strong electrolytes, and affects the characteristics of anion-exchange membranes (AEMs) [40]. This means that the concentration polarization, which is affected by PEF, may not develop according to the generally accepted scenario. At the same time, the effect of PEF on mass transfer, energy consumption, and fouling was studied mainly for the ED of strong electrolyte solutions [41,42] or specific multicomponent solutions [14,26,43,44]. Therefore, the utility of PEF in relation to the ED processing of phosphate-containing solutions is still debatable.

This study was performed to determine the potential of PEF to improve the efficiency of ED recovery of P^V^ compounds from moderately dilute sodium dihydrogen phosphate solutions and then evaluate the role of phosphates on ED performance during treatment of a multicomponent solution that is prone to precipitation. We focused on the anion-exchange membrane that drives phosphate recovery by electrodialysis.

## 2. Materials and Methods

### 2.1. Membranes

Heterogeneous ion-exchange membranes MA-41P (membranes under study), as well as MK-40 and MA-41 (auxiliary membranes) were used. All membranes are manufactured (Shchekinoazot, Pervomaisky, Russia) by hot rolling from low-pressure polyethylene powder and milled ion-exchange resins. Polystyrene regularly crosslinked with divinylbenzene is the polymer matrix of all ion-exchange resins. Nylon reinforcement fabrics are pressed into this composite on both sides of the membranes. The main characteristics of the membranes are presented in Table 1.

### 2.2. Solutions

Series 1 experiments were carried out with NaH_2_PO_4_ phosphate solutions: 0.03 M solution was desalted in a laboratory ED cell; 0.02 M solution was used to measure the current-voltage curves.

Series 2 experiments were carried out with a multicomponent solution in the diluate loop (circuit). This solution imitated the sweet whey of the dairy industry and contained 0.045 M Na_x_H_(3−x)_PO_4_, 0.02 M KCl, 0.045 M KOH, 0.028 M CaCl_2_, and 0.012 M MgCl_2_. KOH was added at the stage of solution preparation in order to achieve pH of 6.0 ± 0.1. 0.02 M KCl solution circulated in other loops of the laboratory ED cell.

Solutions were prepared from distilled water (electric conductivity of 1.1 ± 0.1 S cm^−1^; pH 5.5 at 25 °C) and the analytical grade salts (Vekton, St. Petersburg, Russia).

It is known that species of orthophosphoric acid (as well as other polybasic acids) are involved in protonation–deprotonation reactions. Therefore, their electric charge depends on pH value. Our calculations using the equilibrium acid dissociation constants for the 1st, 2nd, and 3rd stages (see Supplementary Materials, SM) resulted in 99.4% of H_2_PO_4_^−^, 0.25% of H_2_PO_4_^2−^, and 0.35% of H_3_PO_4_ in Series 1 solutions. We designate them as NaH_2_PO_4_, taking into account the predominant content of singly charged anions.

The solution used in Series 2 contains 94.03% of H_2_PO_4_^−^, 5.96% of H_2_PO_4_^2−^, and 0.01% of H_3_PO_4_.

### 2.3. Electrodialysis Desalination of Phosphate-Containing Solutions

**Figure 1:**
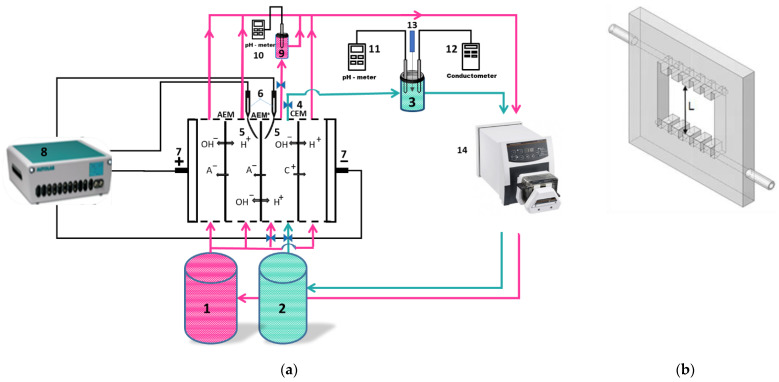
Scheme of the experimental setup (**a**) and a plexiglass frame with special comb guides (**b**) to ensure the laminar hydrodynamic solution flow. Explanations are in the text.

Figure 1 shows a scheme of the ED setup and laboratory cell used in the experiments. Select parameters of this cell and experimental conditions are presented in Table 2.

The membrane stack of the four-compartment ED flow cell (Figure 1a) consisted of the studied anion-exchange membrane (AEM*), as well as auxiliary anion-exchange (AEM) and cation-exchange (CEM) membranes. They were separated by plexiglass frames with special solution input and output devices (Figure 1b) which provided a laminar hydrodynamic regime in each compartment. AEM* and CEM formed a desalination compartment. 

The electric current was set by platinum polarizing electrodes (7) connected to a power source, electrochemical complex Autolab PGSTAT100 (Metrohm, Utrecht, the Netherlands) (8). The potential drop was recorded using Luggin capillaries (5) located at a distance of 0.8 mm from the AEM* surfaces. Each capillary was connected to a microreservoir with a Ag/AgCl electrode (Gomel, Belorussia) (6). Autolab PGSTAT100 PGSTAT100 recorded the potential drop.

The measurement of the AMX* membrane current–voltage curves was carried out at a current sweep rate of 0.02 mA s^−^^1^. A combined pH electrode in a flow microreservoir (9) was controlled the pH at the outlet of the desalination compartment using an Expert 001 pH meter (LLC “Ekoniks expert”, Russia) (10). In Series 1, CVC was obtained by supplying a 0.02 M NaH_2_PO_4_ solution from tanks (1) and (2). The solution returned to the same tank after passing through the compartments of the ED cell. The volume of the solution in the tanks was 5 dm^3^; therefore, the electrolyte concentration and pH changed negligibly during the experiment. After obtaining the CVC, the solution in the tank (2) was replaced with a fresh one. The diluate loop was switched to a tank (3) using valves (4) and filled with 0.03 M NaH_2_PO_4_ solution. The volume of this solution was 0.1 dm^3^. It was selected in such a way that the concentrations of the components of the desalinated solution decreased at a rate of less than 1% per minute in order to provide quasi-equilibrium ED conditions [50]. The experimental setup did not contain special devices to prevent the dissolution of atmospheric carbon dioxide in the feed solutions. Previous studies [50] have shown that the rate of this dissolution is approximately 5∙10^−^^8^ mol dm^−^^3^ s^−l^ (at pressure 1 atm, temperature 25 °C, and pH 7). Therefore, a significant contribution of carbonic acid species to mass transfer can be expected only if the diluate concentration becomes 0.5 mM or less.

The combined electrode for pH measurement, connected to the pHM120 MeterLab pH meter (11), and the conductometric cell, connected to the Expert-002 conductometer (12), were in the tank (3). They served for periodic (every 10 min) measurement of pH, and conductivity of the solution. A 0.1 M NaOH was supplied into the tank (3) through a thin capillary (13) to maintain a constant pH value in the diluate loop. A peristaltic pump (Heidolph, Schwabach, Germany) (14) was used to pump all solutions. Electrodialysis was carried out in batch mode. The experiment was stopped when the conductivity of the desalinated solution, *κ*, decreased by 40% compared with the initial value. The previously obtained calibration curve *κ = f(c)* was used to determine the concentration of NaH_2_PO_4_ in the desalinated solution at each time point. 

The transport numbers $T_{i}^{AEM}$ of orthophosphoric acid anions (i) in the anion-exchange membrane were found under the assumption that only two types of phosphorus-containing species presented simultaneously in AEM. These were H_2_PO_4_^−^ and HPO_4_^2−^ in the case of 4.6 < pH < 9.8, as well as HPO_4_^2−^ and PO_4_^3−^ in the case of 9.8 < pH < 13.0. The following equations were used for calculations [38]:

(1) $$j_{H_{2}PO_{4}{}^{-}}^{s}=-\frac{\bar{V}}{S}\frac{dC_{NaH_{2}PO_{4}}}{dt}$$

(2) $$i_{H_{2}PO_{4}{}^{-}}^{AEM}=2Fj_{H_{2}PO_{4}{}^{-}}^{s}-i$$

(3) $$i_{HPO_{4}{}^{2-}}^{AEM}=2\left(i-Fj_{H_{2}PO_{4}^{-}}^{s}\right)$$

(4) $$i_{HPO_{4}{}^{2-}}^{AEM}=2\left(3Fj_{H_{2}PO_{4}{}^{-}}^{s}-i\right)$$

(5) $$i_{PO_{4}{}^{3-}}^{AEM}=3\left(i-2Fj_{H_{2}PO_{4}{}^{-}}^{s}\right)$$

(6) $$T_{i}^{AEM}=i_{i}^{AEM}/i$$

The current efficiency was calculated using the equation:

(7) $$\eta =T_{H_{{}_{2}}PO_{4}^{-}}+T_{HPO_{4}^{2-}}/2+T_{PO_{4}^{3-}}/3=(i_{H_{{}_{2}}PO_{4}^{-}}+1/2i_{HPO_{4}^{2-}}+1/3i_{PO_{4}^{3-}})/i_{tot}$$

Here $j_{H_{2}PO_{4}{}^{-}}^{s}$ is the flux of singly charged orthophosphoric acid anions recovered from desalinated solution (denoted by index s); $i_{H_{2}PO_{4}{}^{-}}^{AEM}$,$i_{HPO_{4}{}^{2-}}^{AEM}$, and $i_{PO_{4}{}^{3-}}^{AEM}$ are the current densities as well as $T_{H_{2}PO_{4}{}^{-}}^{AEM}$,$T_{HPO_{4}{}^{2-}}^{AEM}$ and $T_{PO_{4}{}^{3-}}^{AEM}$, which are the transport numbers of single-, double-, and triple-charged orthophosphoric acid anions in the membrane (denoted by index AEM); $\bar{V}$ is the volume of the solution in the diluate loop; *S* is the polarizable membrane area; $\frac{dC_{NaH_{2}PO_{4}}}{dt}$– is the rate of decrease in electrolyte concentration ($C_{NaH_{2}PO_{4}}$) in the diluate loop; *t* is the time; *i_tot_* is the total current density; and F is the Faraday constant. Multipliers «2» and «3» in Equations (2)–(5) and (7) correspond to electric charges $z_{HPO_{4}{}^{2-}}$ and $z_{PO_{4}{}^{3-}}$, respectively. Index *i* in Equation (6) denotes the anions of ortophosphoric acid. The derivation of these equations is presented in Supplementary Materials.

The degree of desalination, $\gamma_{D}{}^{{}_{}}$, and the degree of removal of component *i*, $\gamma_{i}$ were found by the equations:

(8) $$\gamma_{D}=\frac{\kappa^{0}-\kappa^{t}}{\kappa^{0}}$$

(9) $$\gamma_{i}=\frac{c_{i}^{0}-c_{i}^{t}}{c_{i}^{0}}$$

where the indices «0» and «t» correspond to the characteristics of the desalinated solution at the beginning and after a given time, respectively, of electrodialysis.

The theoretical limiting current was calculated using the conventional Leveque equation [51] (NaH_2_PO_4_ solutions) or according to the modified Leveque equation [38] (multicomponent solution), presented in Supplementary Materials.

In Series 2, the desalination compartment was fed from the tank (3) with a multicomponent solution. All other compartments of the ED cell were supplied from the tank (2) with a 0.02 M KCl solution. Experiments were stopped when the conductivity of the desalinated solution decreased by 50% compared with its initial conductivity. The concentrations of every component in a multicomponent solution before and after ED were determined by ion-exchange chromatography using DIONEX CS 16 and AS 19 chromatographic columns to separate mixtures of cations and anions, respectively. CVC were obtained before and after desalination of the solution by ED.

In both Series 1 and Series 2, electrodialysis was carried out in current modes CC and PEF.

**Figure 2:**
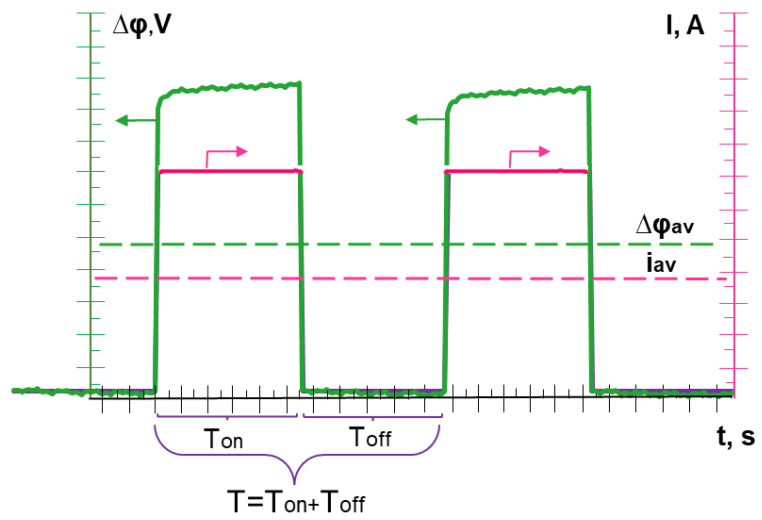
The applied current densities and the corresponding potential drops vs. time in the PEF mode. The dotted lines indicate the average values of *i_av_* and *Δφ_av_* for the period.

PEF is an alternation of periods, each of which consisted of a constant current density pulse lapse of duration *Ton* and a pause lapse (i = 0) of duration *Toff*. PEF frequency was determined as *f* = 1/*T*, where *T = Ton + Toff* (Figure 2). 

Values of the duty cycle of PEF, α, were found as: *α = T_on_/T*. The average value of the current density during the application of PEF was determined as:

(10) $$i_{av}=i\alpha$$

where *i* is the current density at the pulse lapse (during *T_on_*).

The average value of the potential drop over the period was calculated by the equation:

(11) $$\Delta \varphi_{av}=\frac{1}{T}\int_{0}^{t}\Delta \varphi dt$$

The number of electric charges transported, *Q*, and energy consumption, *W*, spent on the desalination of the solution was determined as:

(12) $$Q=I_{av}t$$

(13) W=∫I(t)Δφ(t)dt

where *I_av_* is the average current intensity over the period. The value of *W* was calculated using Equation (13), according to the dependences of *I(t)* and *Δφ(t)* measured using the Autolab PGSTAT100 electrochemical complex. Integration in accordance with Equation (13) was carried out from the beginning of the ED process (*t* = 0) to the time *t = t′*, when the desired (40% or 50%) degree of desalination was reached.

### 2.4. Scaling Characterization 

Scanning electron microscopy (SEM) combined with X-ray diffraction analysis (XRD) was used to study scaling during electrodialysis. We applied a JEOL JSM-7500F (JEOL Ltd., Tokyo, Japan) scanning electron microscope with an INCA X-sight energy dispersive attachment.

## 3. Results

### 3.1. Desalination of NaH_2_PO_4_ Solution 

Desalination of 0.03 M NaH_2_PO_4_ solution (Series 1) was carried out at current modes, the parameters of which are summarized in Table 3.

In each of the experiments, the average current density (*i_av_*) was maintained constant and equal to the current density in the CC mode (*i_CC_*): *i_av PEF1_* = *i_CC1_* = 3.0 mA cm^−^^2^ and *i_av PEF2_* = *i_CC2_* = 3.5 mA cm^−^^2^.

**Figure 3:**
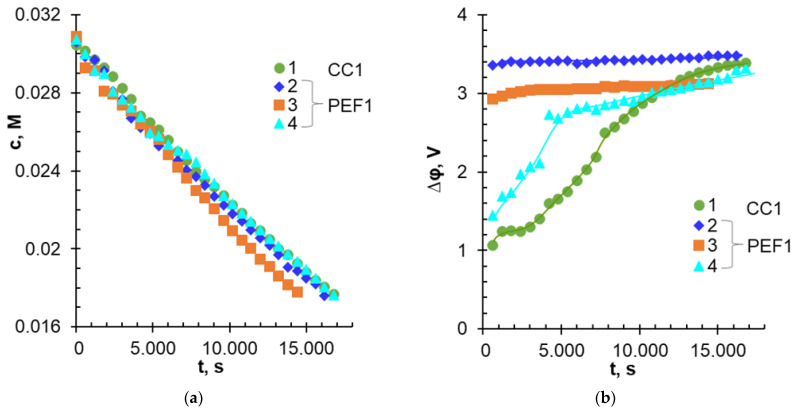
Changes in the NaH_2_PO_4_ concentration in the diluate loop (**a**) and the potential drop between Luggin capillaries (**b**) vs. the duration of 0.03 M NaH_2_PO_4_ solution desalination at *i_av_ = i_CC1_* = 3 mA cm^−^^2^. Other parameters of the current modes are given in Table 3.

**Figure 4:**
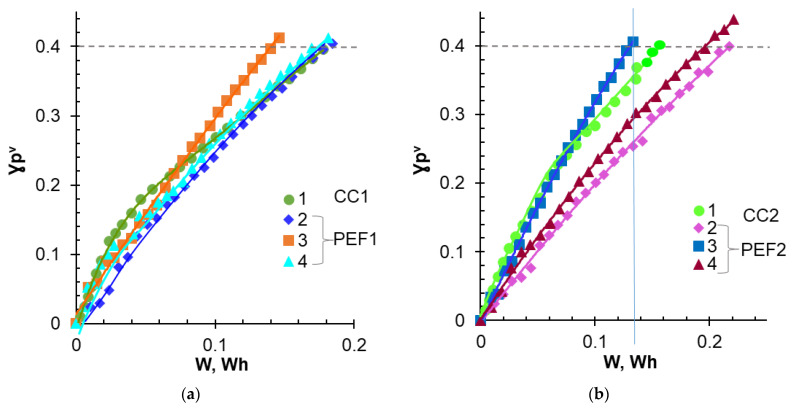
Dependences of the degree of P^V^ removal upon the energy consumption for average current densities equal to 3.0 (**a**) and 3.5 (**b**) mA cm^−2^. Other parameters of the current modes are given in Table 3. The dotted line corresponds to $\gamma^{P^{V}}$ = 40%.

Dependences of the NaH_2_PO_4_ concentration in the diluate loop and the potential drop between Luggin capillaries upon the duration of NaH_2_PO_4_ solution desalination are shown in Figure 3. These data were obtained at average current densities equal to 3 mA cm^−^^2^. Figure 4 shows the degree of P^V^ removal from the diluate vs. the energy consumption for average current densities of 3.0 and 3 mA cm^−^^2^. Table 4 summarizes the characteristics of the ED process at the degree of removal of 40% ($\gamma^{P^{V}}$ = 40%).

In Experiment 1 *(i_CC1_ = i_av PEF1_* = 3.0 mA cm^−^^2^), the desalination rate ($\frac{dC_{NaH_{2}PO_{4}}}{dt}$) of NaH_2_PO_4_ solution weakly depended on the applied current mode up to a concentration of 0.02 M (Figure 3a). Further duration of the experiment was accompanied by an increase in the desalination rate in the PEF1-3 mode compared with CC1, PEF1-2, and PEF1-4 modes. The dependencies *Δφ–t* (Figure 3b) had a complex shape. In the case of CC1, *Δφ* value increased with time. This growth slowed down significantly only toward the end of the experiment, when the solution desalination approached 40%. In PEF1 modes, the curves *Δφ–t* also had sections of fast and slow increase of the potential drop. The time for the curve to reach a plateau increased in the order: PEF1-3 < PEF1-4 < PEF1-2, and the values of *Δφ* became close for all current modes at $\gamma^{P^{V}}$ > 40%.

The dependencies of $C_{NaH_{2}PO_{4}}$*–t* and *Δφ–t* in Experiment 2 (*i_CC2_ = i_av_*_PEF2_ = 3.5 mA cm^−^^2^) were similar to those described above. The difference lies in the decreasing of the time required to reach $\gamma^{P^{V}}$= 40% in the CC2 and PEF2-3 modes to 1280 s and 13200 s, respectively, against 16800 s (CC1) and 1300 s (PEF1-3). Moreover, final *Δφ* values depended more strongly on the current mode. They were equal to 2.93 V (CC2); 2.65 V (PEF2-3); 3.5 V (PEF2-4); and 3.6 V (PEF2-2).

In both experiments, PEF modes with α = 1/2 and f = 5 Гц (PEF1-3, PEF2-3) provided lower energy consumption compared with other modes when the desired value of $\gamma^{P^{V}}$ exceeded 22 ± 2% (Figure 4), and the profit in energy consumption was higher in the case of PEF1-3, compared with PEF2-3 mode (Figure 4 and Table 4). On the contrary, energy consumption increased significantly in PEF2-2 and PEF2-4 modes compared with PEF1-2 and PEF1-4 modes. Thus, a relatively small increase in i_av_ caused significant differences in the behavior of the membrane system.

**Figure 5:**
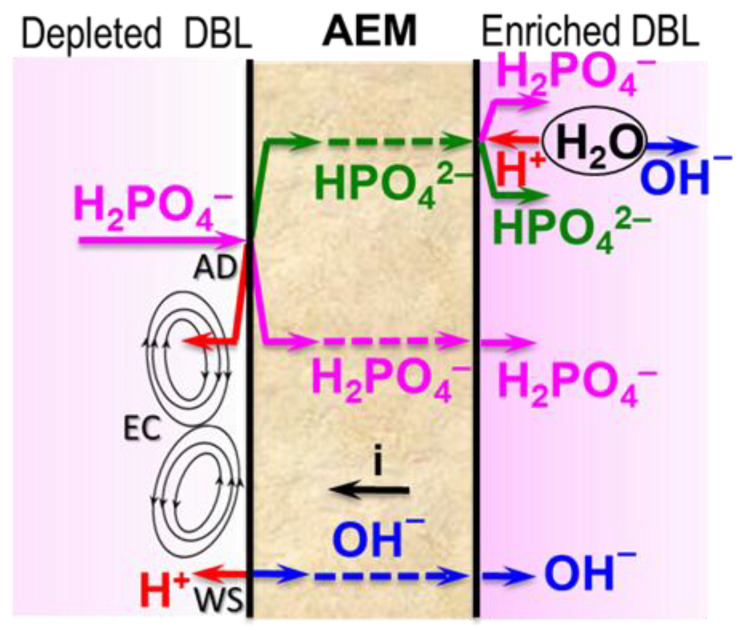
Scheme of phenomena affecting phosphate transfer in ED desalination of NaH_2_PO_4_ solution. Explanations are in the text.

These differences can be explained by drawing on the results of previous studies [38]. According to them, the current efficiency in the case of phosphate-containing solutions is governed by three phenomena (Figure 5): “acid dissociation” (AD), water splitting (WS), and electroconvection (EC). “Acid dissociation” and water splitting mainly reduce the current efficiency. On the contrary, electroconvection enhances it.

WS is the generation of H^+^, OH^−^ ions at the AEM/depleted solution interface involving fixed groups of membranes [52]. EC is the mixing of the depleted solution at the AEM surface due to the appearance of vortex structures [53]. Both of these phenomena occur in overlimiting current modes. Their nature is identical for strong electrolytes (NaCl) and for NaH_2_PO_4_ solutions. AD is the deprotonation of H_2_PO_4_^−^ with the formation of a doubly charged anion HPO_4_^2−^ when a singly charged orthophosphoric acid anion enters the AEM [38]. The proton is excluded into solution due to the Donnan effect [54]. HPO_4_^2−^ anion is transported by the electric field to the AEM/enriched solution interface and participates in the protonation–deprotonation reactions. The products of these reactions are OH^−^ and H_2_PO_4_^−^ anions. This phenomenon is specific for the electrodialysis of solutions of phosphates and other salts of polybasic acids [38,55,56,57]. AD occurs at any current but increases at *i≈ i_lim_^Lev^* due to the enhancement of the Donnan exclusion of protons. The reason for the intensification of the Donnan effect is a dramatic decrease in the concentration of NaH_2_PO_4_ in the depleted solution near the surface of MA-41P [54]. If *i* < 2.2*i_lim_^Lev^*, proton generation in phosphate-containing membrane systems is due mainly to the AD mechanism [38]. This current corresponds to the saturation of the membrane with doubly charged HPO4^2−^ anions, the establishment of a constant proton generation rate by the AD mechanism, as well as the beginning of intense WS and nonequilibrium EC [38]). If *i* > 2.2*i_lim_^Lev^*, both AD and WS are involved in proton generation [38]. Therefore, the concentration of protons in the near-membrane depleted solution increases. These protons reduce the extended space charge density at the AEM/depleted solution interface, which leads to a suppression of EC in the CC [39] and PEF [58] modes. 

**Figure 6:**
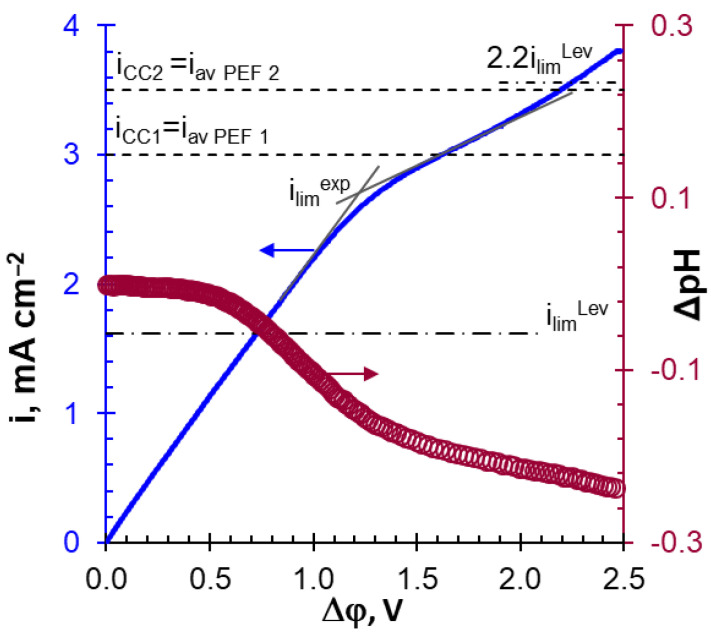
Current–voltage curves of the MA-41P membrane and the corresponding dependence of the pH difference at the inlet and outlet of the ED desalination compartment upon the potential drop. Data obtained in 0.02 M NaH_2_PO_4_ solution. The dashed-dotted line is the theoretical limiting current calculated according to Equation (10). The dotted lines indicate the average applied current densities in Experiments 1 and 2.

Estimates using Equation (10) produced *i_lim_^Lev^* values of 2.43 mA cm^−^^2^ for 0.03 M NaH_2_PO_4_ solution. At 40% degree of desalination, *i_lim_^Lev^* will drop to 1.46 mA cm^−^^2^. Thus, it can be expected that the values of the average current in Experiment 1 will not exceed the value 2.2*i_lim_^Lev^* for any current mode. In Experiment 2, the value 2.2*i_lim_^Lev^* can be exceeded at desalinated solution concentrations less than 0.02 M. In practice, the WS begins to participate in the generation of protons at lower current densities. Essentially, Equation (10) is valid for homogeneous membranes, all surfaces of which conduct an electric current. For these membranes, the theoretical value of 2.2 *i_lim_^Lev^* was close to the *i_lim_^exp^* [38], which was determined from the experimental CVC, as shown in Figure 6. A heterogeneous MA-41P membrane had only 35% of the conductive surface (Table 1). Therefore, the current density at the conductive areas of the heterogeneous membrane was higher in comparison with a homogeneous one [48,59]. The enhancement of the concentration polarization led to a decrease in the experimental limiting current compared with the theoretical. Changes in the slopes of the *ΔpH–Δφ* curve implicitly indicate an intensification of AD and the onset of WS (as well as significant EC) at *i* ≈ 0.5*i_lim_^exp^* and *i* ≈ *i_lim_^exp^*, respectively (Figure 6). 

Note that the average current densities applied in Experiments 1 and 2 became higher than the critical *i_lim_^exp^* current at a concentration of desalinated solution of 0.02 M or less (Figure 6). At the CC1 mode, the applied current density of 3.0 mA cm^−^^2^ corresponded to the upper limit when the AD mechanism dominated. At the CC2 mode (*i* = 3.5 mA cm^−^^2^), the contribution of WS and EC to mass transfer should be more significant. According to Equation (11), the current density (*i*) during the PEF pulse lapse increases with a decrease in the duty cycle of the PEF (α) compared with the CC mode. This means that during the pulse lapse at the PEF mode, one can expect changes in the contributions to the mass transfer of the WS and EC mechanisms compared with CC. In addition, the concentration profiles of the components of the solution, which are formed near the surfaces of the MA-41P during the pulse, will be more significantly blurred with a decrease in the duty cycle due to an increase in the duration of the pause.

Indeed, a more significant contribution of WS to proton generation in Experiment 2 compared with Experiment 1 was expressed in an increase in the number of protons entering the desalinated solution during ED, as well as in an increase in the transport numbers of HPO_4_^2−^ anions in the MA-41P membrane under all current modes (Table 4). At the CC1 and CC2 modes, this difference was minimal and close to the experimental errors, because the applied currents were relatively close. The greatest differences in the number of generated protons were observed in the PEF1-3 and PEF2-3 (α = 1/4, *f* = 5 Hz) modes, when the current exceeded the *i_lim_^exp^* value during the pulse lapse by 4.4 and 5.2 times, respectively. In the case of PEF1-4 and PEF2-4 (α = ¾, *f* = 5 Hz) modes, this excess was only 1.5 and 1.7 times, respectively. However, the duration of the impulse, which contributed to the accumulation of protons in the desalinated solution, was maximal. The minimal acidification of the desalinated solution and the transformation in MA-41P of H_2_PO_4_^−^ into HPO_4_^2−^ anions were ensured in the PEF1-3 and PEF2-3 (α = 1/2, *f* = 5 Hz) modes. These modes provided a decrease in energy consumption by 20 ± 2% compared with the CC1 and CC2 modes during desalination of NaH_2_PO_4_ solutions by 40%. We assume that this advantage is due to the optimal relationship between the applied current during the PEF pulse and the duration of the pulse and pause. Apparently, the applied current ensured the development of EC, which mixed the solution at the MA-41P surface facing the desalination compartment. Moreover, EC vortices do not have enough time to “dissipate” completely during the pause lapse, contributing to the enhancement of EC during the next current pulse. EC mixing promotes an increase in the surface concentration of NaH_2_PO_4_ solution. An increase in this concentration reduces the Donnan exclusion of protons from the membrane [54] and their negative effect on EC due to a decrease in the space charge density [60] at the AEM/depleted solution interface.

As shown earlier [39,58], it is precisely the more intense proton generation that causes the suppression of EC in phosphate-containing solutions as compared with solutions of strong electrolytes. Nevertheless, this study allowed us to conclude that the contribution of EC to the mass transfer of phosphates remains quite significant if the *i_lim_^exp^* value is not exceeded by more than 2.0–2.5 times. It is known [61] that solution dilution enhances the EC. Apparently, when the concentration of the desalted solution is less than 0.02 M, the EC vortices remain sufficiently large both during the pulse and during the pause. More intense electroconvective delivery of electrolyte to the surface of MA-41P helps to reduce energy consumption in the PEF1-3 and PEF2-3 compared with other modes.

The complex dependences of the measured potential dropped upon the duration the electrodialysis (Figure 3b), most likely caused by a change in the composition of the depleted near-membrane solution. An increase in the potential drop with an increase in the duration of ED was due to an increase in the resistance of the depleted solution between the Luggin capillary and the membrane surface. The achievement of constant values of the potential drop in dilute solutions was caused by the stabilization of the sizes of EC vortices and the rate of proton generation due to AD and WS. The relationship between H_2_PO_4_^−^, HPO_4_^2−^, PO_4_^3−^, or OH- anion concentrations in AEM had little effect on the value of *Δφ*, as was shown in [39]. 

### 3.2. Desalination of a Multicomponent Solution

*ED performance in* *CC and PEF modes*

The PEF mode (*α* = 1/2, 5 Hz) was used for ED desalination of the multicomponent solution containing 0.045 M Na_x_H_(3−x)_PO_4_, 0.02 M KCl, 0.045 M KOH, 0.028 M CaCl_2_, and 0.012 M MgCl_2_ (pH 6.0 ± 0.1). These parameters of the PEF provide the maximum advantage over the CC mode for the desalination of the NaH_2_PO_4_ solution, as was shown in Section 3.1. Two CC modes were studied for comparison (Table 5). The applied current density in the CC1 mode (*i_CC_*_1_) was equal to the average current density (*i_av PEF_*) in the PEF mode (4 mA cm^−^^2^). In the CC2 mode, *i_CC2_* was equal to the applied current density in the PEF mode during the pulse.

**Figure 7:**
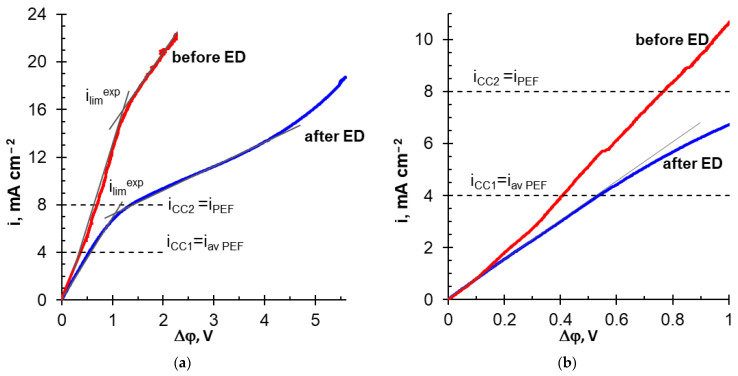
Current–voltage curves of the MA-41P membrane before and after ED desalination of the multicomponent solution (**a**) and initial sections of these curves (**b**).

The applied current densities in these modes are marked with dotted lines in Figure 7. The same figure shows the CVCs of MA-41P and adjacent layers of depleted and enriched solutions. The curve obtained in a solution with pH 6.0 ± 0.1 and the composition of 0.045 M Na_x_H_(3−x)_PO_4_, 0.02 M KCl, 0.045 M KOH, 0.028 M CaCl_2_, and 0.012 M MgCl_2_, is denoted as “before ED”. The curve obtained after desalination of the solution by 50% in the PEF mode is designated as “after ED”. The applied current densities (*i_CC1_, i_CC2_, i_PEF_, i_av PEF_*) did not exceed *i_lim_^exp^* value during the experiments.

Note that the multicomponent solution contains phosphoric acid anions, the fraction of which in the transfer of electric charge (in equivalent) is 31%, while the fraction of Cl^−^ anions is 69%. Therefore, a smaller contribution of «acid dissociation» to the ED desalination of this multicomponent solution is expected in comparison with NaH_2_PO_4_ solution (Section 3.1). At the same time, the presence of this mechanism is evidenced by two inclined plateaus on the “before ED” CVC. Apparently, the first plateau (observed between potential drops from 0.02 to 0.04 mV) characterizes diffusion limitations in the delivery of phosphoric acid anions from the bulk of the depleted solution to the surface of MA-41P membrane, as in the case of the NaH_2_PO_4_ solution. The second inclined plateau (*Δφ* > 2 V) corresponds to diffusion limitations in the delivery of Cl^−^ anions. The plateau caused the saturation of MA-41P membrane with doubly charged HPO_4_^2−^ anions is not identified on the “before ED” CVC due to quite high concentration of Cl^−^ anions at the membrane surface. «After ED» CVC has only one pronounced inclined plateau in the vicinity of *i_PEF_* (Figure 7).

**Figure 8:**
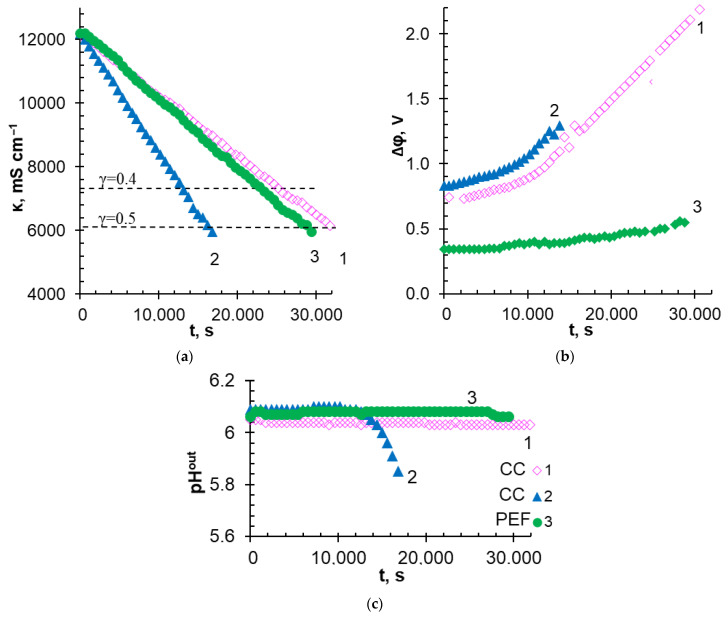
Dependences of the conductivity of the desalinated multicomponent solution (**a**), the potential drop on the MA-41P membrane and adjacent depleted and enriched solution layers (**b**), as well as the pH values of the solution at the outlet of the desalination compartment (**c**) upon the duration of the electrodialysis in the CC1 (Curve 1), CC2 (Curve 2), and PEF (Curve 3) modes.

The conductivity of the desalinated multicomponent solution, the potential drop on the MA-41P membrane and adjacent depleted and enriched solution layers, as well as the pH of the solution at the outlet of the desalination compartment vs. ED duration in the CC1, CC2, and PEF modes, are shown in Figure 8. Table 6 summarizes some characteristics of the ED process.

In general, the results obtained for the CC modes, as expected, are in a good agreement with the numerous studies of conventional ED of strong electrolytes summarized by Strathmann [26,62]. A linear dependence of the decrease in the conductivity of the desalinated solution upon the ED duration was observed (Figure 8a). With an increase in current density by two times (CC2 mode), the rate of solution demineralization increased by approximately the same times (Table 6), and the time required for a twofold decrease in the solution conductivity decreased in the same manner (Figure 8a), compared with the CC1 mode. These positive achievements from the use of the CC2 mode were accompanied by an increase in energy consumption by 1.4 times (Table 6) due to an increase in the potential drop. At the beginning of the ED, the *Δφ* values corresponded to those found from the CVC for the current densities applied in the experiment (Figure 7). As expected, the potential drop was approximately twice as high in the CC2 mode as in the CC1 mode (Figure 8b). This ratio between the potential drops in the CC1 and CC2 modes remained approximately the same up to solution desalination by approximately 30%. Further desalination of the solution caused more intense proton generation in the CC2 mode (Figure 8c). Enrichment of the depleted solution with protons led to a decrease in its resistance. As a result, a potential drop was reduced in CC2 mode compared with CC1. Indeed, intensive acidification of the desalinated solution in the CC2 mode was observed approximately 10000 s after the start of ED (Figure 8c), when γ_D_ was reached approximately 30%. Apparently, the main cause of acidification of the depleted near-membrane solution was WS, which began on the conductive areas of the MA-41P surface at *i < i_lim_^exp^* [48]. Note that the desalinated multicomponent solution had a high buffer capacity. Therefore, it can be assumed that the enrichment of the near-membrane solution with protons began earlier than can be observed by measuring the pH of the solution at the outlet of the desalination compartment.

The use of PEF mode led to a significant decrease in the recorded potential drop compared with the CC2 mode (Figure 8b). Directly after the beginning of ED in the PEF mode, the *Δφ* value was almost equal to that recorded in the CC1 mode. This result was quite expected, because the *i_av_* value in PEF and CC1 modes was the same. With an increase in the duration of the ED, the difference in potential drop in the CC1 and PEF modes became increasingly more significant. By the end of the experiment, *Δφ* (PEF) turned out to be almost 35% less than *Δφ* (CC1). The demineralization rate of a multicomponent solution increased compared with the CC1 mode (Figure 8a and Table 6). At the same time, the pH value of the desalinated solution remained practically unchanged during the experiment in the PEF mode (Figure 8c). Energy consumption for desalination of the multicomponent solution by 40% was reduced by 4% and 25% compared with CC1 and CC2 modes, respectively.

In the case of $\gamma_{D}$ = 50%, the savings in energy consumption from the use of PEF increases to 6% and 28% compared with CC1 and CC2, respectively. It should be noted that a twofold decrease in the conductivity of the treated solution was achieved for all applied current modes due to the recovery of approximately 60 ± 4% of Cl^−^ anions, 40 ± 4% of K^+^, Na^+^ cations, and 60 ± 4% of Ca^2+^ cations as well as 20 ± 4% of anions that contain P^V^ (Table 6). This degree of removal of substances containing P^V^ is in good agreement with the data presented by Lemay et al. [26] for the demineralization of sweet milk whey with a mineral composition close to the solution used in this research. This result was achieved despite the fact that in [26], homogeneous IEMs (Astom, Japan) were used, while we used a heterogeneous anion-exchange membrane. Note that Lemay et al. achieved a 20% reduction in power consumption, while in our case the profit was 28% in PEF mode compared with CC1 mode. This difference seems to be because our estimates were made only for the AEM and adjacent layers of depleted and enriched solutions. At the same time, Lemay et al. studied the membrane stack of the electrodialyzer, which contained non-conductive spacers. 

The reasons for the reduction in energy consumption when using the PEF mode compared with the CC modes have already been discussed in Section 3.1. Note that a sufficiently high initial concentration of a multicomponent solution can contribute to the development of gravitational convection [63,64] in addition to the already discussed AD, WS, and EC. However, the effect of the PEF mode on gravitational convection has not yet been studied.

It is important to note that the degree of Mg^2+^ cations removal in the experiments turned out to be different: 63 ± 4% (CC1), 48 ± 4% (CC2), and 44 ± 4% (PEF). The reasons for this difference will be discussed below.

*Scaling*

As noted in the Introduction, ED processing of solutions containing Ca^2+^ и Mg^2+^ ions is often accompanied by scaling [11,12]. Scaling forms on the surfaces of both CEM and AEM. Precipitation occurs due to an excess of local solubility product of partially soluble salts near the membrane surface and/or alkalization of the near-membrane solution. 

**Figure 9:**
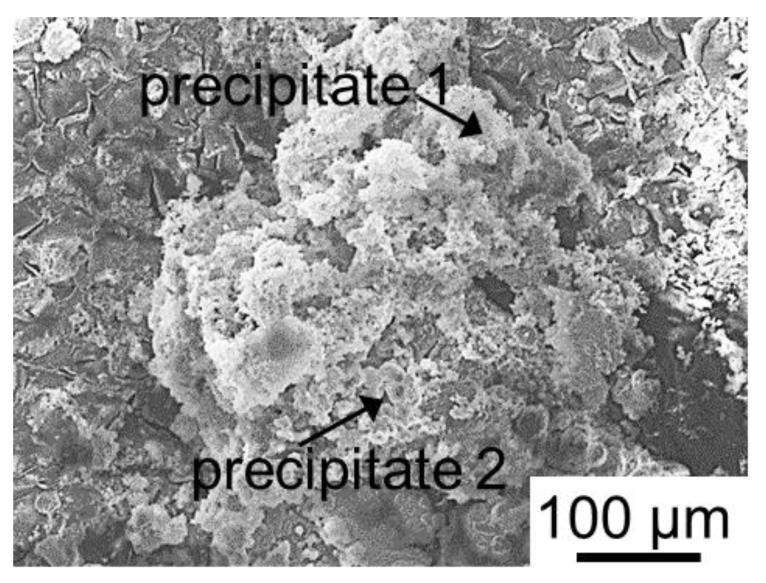
SEM images of the MC-40 cation exchange membrane surface facing the desalination compartment after ED demineralization of a multicomponent solution in CC1 mode.

In the case of the heterogeneous MK-40 cation-exchange membrane, which had only 15 ± 3% of the conductive surface (Table 1), the largest amount of scaling was found in the CC1 mode (Figure 9), which primarily contained magnesium (40 wt%) and oxygen (45 wt%) as well as some calcium (9 wt%) and phosphorous (6 wt%). It is known [65] that magnesium cations participate in protonation–deprotonation reactions, enhancing WS on the surface of cation-exchange membranes. We assume that WS began long before the experimental limiting current which equaled 9.4 mA cm^−^^2^ (while for MA-41P, it was 18.1 mA cm^−^^2^) for the initial multicomponent solution and an approximately twofold decrease at $\gamma_{D}{}^{{}_{}}$ = 50%. A local increase in pH at the KU-2-8 resin/depleted solution interface caused the formation of Mg(OH)_2_ in brucite form [66] and possibly some portlandite, Ca(OH)_2_ (precipitation 1, Figure 9), as well as large drop-shaped crystals of hydroxyapatite, Ca_5_(PO_4_)_3_(OH) (precipitate 2, Figure 9). On the contrary, acidification of the desalinated multicomponent solution in the CC2 mode (Figure 8c) or a decrease in the duration of electric current exposure in the PEF mode, apparently, contributed to the partial dissolution of Mg(OH)_2_. As a result, the loss of Mg^2+^ from the treated solution in the CC2 and PEF modes decreased compared with the CC1 mode (Table 6). 

**Figure 10:**
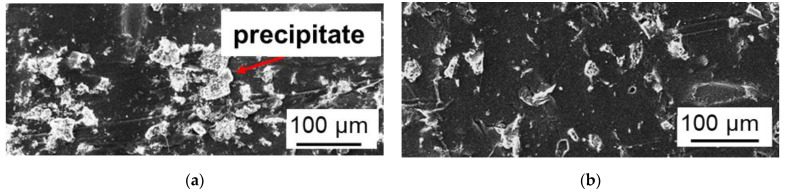
SEM images of the MA-41P membrane surface facing the desalination compartment after ED demineralization of a multicomponent solution in CC2 (**a**) and PEF (**b**) modes.

**Figure 11:**
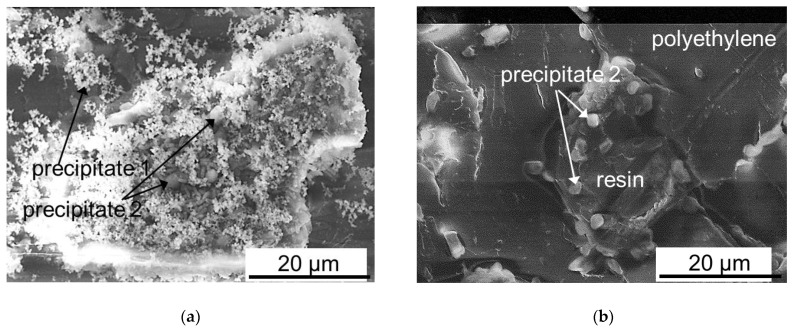
SEM images of mineral precipitates on granules of anion-exchange resin protruding on the surface of MA-41P after ED demineralization of a multicomponent solution in CC2 (**a**) and PEF (**b**) modes.

In the case of MA-41P, the most noticeable scaling was visualized after ED desalination in the CC2 mode (Figure 10a and Figure 11a). The precipitate layer covered almost the conductive surface of the MA-41P membrane, forming large drop-shaped and smaller elongated crystals (Figure 10a). The precipitate contained 48 wt% of O, 12 wt% of P, 3 wt% of Cl, 5 wt% of Mg, 6 wt% of Ca, and 3 wt% of K. Such a composition, as well as the shape of the crystals, which were described in [26], allow us to make the following assumption. A small crystals of brucite Mg(OH)_2_ and possibly some portlandite, Ca(OH)_2_ (precipitate 1, Figure 11a), as well as large drop-shaped crystals of hydroxyapatite, Ca_5_(PO_4_)_3_(OH) (precipitate 2, Figure 11a,b) are formed on the grains of the AV-17-2P resin. Precipitation is apparently caused by the high pH inside the anion-exchange material [38], as well as the accumulation of phosphoric acid anions near the MA-41P surface due to the predominant transport of more mobile Cl^−^ anions through the AEM. In addition, the nuclei of brucite and portlandite crystals formed in the depleted diffusion layer near the surface of MC-40 [67] can be delivered to the surface of MA-41P due to convection. The rough surface of heterogeneous MA-41P membrane promotes their deposition.

Only individual drop-like crystals of hydroxyapatite are observed at the boundaries of the anion-exchange resin granules and polyethylene on the MA-41P surface after the ED desalination in the PEF mode (Figure 10b and Figure 11b). Thus, the use of PEF significantly reduces scaling on the surface of heterogeneous AEM. 

## 4. Conclusions

A comparative analysis of mass transfer characteristics and energy consumption was carried out for the electrodialysis (ED) processing of NaH_2_PO_4_ solution and multicomponent solution whey imitating (0.045 M NaxH_(3−x)_PO_4_, 0.02 M KCl, 0.045 M KOH, 0.028 M CaCl_2_ и 0.012 M MgCl_2_, pH 6.0 ± 0.1). Research was carried out in a conventional continuous current mode (CC) and a pulsed electric field (PEF) mode with a frequency of 5 Hz and different duty cycles. A lab-scale ED set-up contained heterogeneous cation-exchange and anion-exchange membranes. Particular attention was paid to the behavior of the anion-exchange membrane MA-41P (LTD Shchekinoazot).

It was shown that the use of PEP is fruitful in the ED recovery of phosphates from moderately dilute solutions.

At 40% desalination of 0.03 M NaH_2_PO_4_ solution, the PEF mode (duty cycle 1/2) provided a 20% reduction in energy consumption compared with the CC mode, when the same average current densities were set. The profit of the PEF mode is achieved mainly due to the suppression of the “acid dissociation” phenomenon which occurred in anion-exchange membranes in phosphate-containing solutions. The use of high current densities at which intense water splitting takes place led to an increase in energy consumption at the PEF with duty cycles 1/4 and 3/4, compared with the modes CC and PEF with duty cycle 1/2. Apparently, the intense generation of H^+^, OH^−^ ions suppressed electroconvection, both in the pulse lapse and the pause lapse.

In the ED desalination of a multicomponent solution, the modes CC1, CC2, and PEF with a duty cycle of ½ were compared. Modes CC1 and PEF were characterized by equal values of average current densities; the CC2 and PEF modes had equal current densities at the moment of the pulse. The PEF mode demonstrates economy in energy consumption by 4% and 25% (the degree of desalination was 40%), as well as 6% and 28% (the degree of desalination was 50%) compared with modes CC1 and CC2, respectively. Apparently, Cl^–^ anions in a multicomponent solution enhanced electroconvection, providing a more significant advantage of PEF compared to the NaH_2_PO_4_ solution.

A scaling of the surface of the MA-41P membrane, facing the desalination compartment was observed in the CC2 mode. The precipitate was localized on the anion-exchange resin grains. The use of PEF mode significantly suppresses the scaling.

## Figures and Tables

**Table 1:** Main characteristics of the membranes.

Membrane	Ion-Exchange Resin	Fixed Groups	Thickness, µm (0.02 M NaCl)	Ion-Exchange Capacity of Wet Membrane, mol g^−1^	Fraction of Conductive Surface, Θ
Anion-exchange membranes					
MA-41P	AV-17-2P [45]	–N^+^(CH_3_)_3_ and a small amount of secondary and tertiary amines	510 ± 50	0.92 ± 0.05 [46]	0.35 ± 0.01 [46]
MA-41	AV-17-8 [45]		450 ± 50 [47]	1.22 ± 0.06 [47]	0.22 ± 0.03 [48]
Cation-exchange membrane					
MK-40	KU-2-8 [45]	–SO_3_^−^	520 ± 20 [47]	1.43 ± 0.08 [47]	0.55 ±0.05 [49]

**Table 2:** Select parameters of the laboratory ED cell and experimental conditions.

Active membrane surface area, *S*, cm^2^	4.00 ± 0.01
Intermembrane distance, *h*, cm	0.66 ± 0.01
Compartment length, *L*, cm	2.00 ± 0.01
Average linear flow velocity, *V*_0_, cm s^−1^	0.40 ± 0.01
Temperature, °C	25 ± 0.2

**Table 3:** Parameters of applied current modes during ED desalination of 0.03 M NaH_2_PO_4_ solution.

	**Designation**		***i*** ***_av_*** **, mA cm^−2^**	***i*** **, mA cm^−2^**	**α**	**f,** **Hz**
Experiment 1	CC1	1	3.0	3.0	1	0
	PEF1	2		12.0	1/4	5
		3		6.0	1/2	5
		4		4.0	3/4	5
Experiment 2	CC2	1	3.5	3.5	1	0
	PEF2	2		14.0	1/4	5
		3		7.0	1/2	5
		4		4.7	3/4	5

**Designation**

***i*** ***_av_*** **, mA cm^−2^**

***i*** **, mA cm^−2^**

**α**

**f,** **Hz**

**Table 4:** Characteristics of ED desalination of 0.03 M NaH_2_PO_4_ solution with 40% P^V^ recovery at average current densities equal to 3.0 (CC1, PEF1) and 3.5 (CC2, PEF2) mA cm^−^^2^.

Designation		* H^+^, mmol	$T_{}^{H_{2}PO_{4}{}^{-}}$	$T_{}^{HPO_{4}{}^{2-}}$	${\eta^{P}}^{V}$	Q, C	W, W h
CC1	1	0.72	0.16	0.84	0.58	191	0.18
PEF1	2 (α = 1/4)	0.95	0.17	0.83	0.59	183	0.18
	3 (α = 1/2)	0.64	0.35	0.65	0.68	162	0.14
	4 (α = 3/4)	0.79	0.16	0.84	0.58	191	0.18
CC2	1	0.74	0.12	0.88	0.56	182	0.16
PEF2	2 (α = 1/4)	1.30	0.13	0.87	0.57	215	0.22
	3 (α = 1/2)	0.68	0.25	0.75	0.63	172	0.14
	4 (α = 3/4)	1.16	0.1	0.9	0.55	197	0.21

* protons generated by the MA-41P membrane and ejected into the desalinated solution.

**Table 5:** Parameters of the current modes during ED desalination of the multicomponent solution.

Designations		*i*, mA cm^−2^	*i_av_*, mA cm^−2^	*α*	*f*, Hz
CC1	1	4	-	1.0	-
CC2	2	8	-	1.0	-
PEF	3	8	4	1/2	5

**Table 6:** Select characteristics of a multicomponent solution ED desalination.

The Current Mode			Demineralization Rate *dκ/dt*, mS cm^−1^ s^−1^	The Degree of Desalination is Equal to 50%							
	i mA cm^2^	i _av_ mA cm^2^		** Q, C	W, W h	The Degree of Removal of Component *i, %					
						Cl^−^	P^V^	Na^+^	K^+^	Ca^2+^	Mg^2+^
CC1	4	-	0.20	500	0.100	57	20	41	42	61	63
CC2	8	-	0.38	537	0.130	58	22	47	37	62	48
PEF	8	4	0.22	470	0.094	61	17	43	41	62	40

* the error in determining the degree of recovery of solution components is ±4%, ** the number of electric charge transported.

## Supplementary Materials

The following are available online at https://www.mdpi.com/article/10.3390/membranes12111107/s1.
