# Supplementary material for: Effect of Pulsed Electric Field on the Electrodialysis Performance of Phosphate-Containing Solutions
Source: Membranes (Basel). 2022 Nov 5;12(11):1107. doi: 10.3390/membranes12111107 (PMC9693851; doi:10.3390/membranes12111107)
Supplement: Supplementary file 1 [file membranes-12-01107-s001.zip › membranes-2005217-supplementary.pdf]

Effect of a pulsed electric field on the mass transfer of phosphates in electrodialysis

Olesya Rybalkina, Ksenia Solonchenko, Daria Chuprynina, Natalia Pismenskaya and Victor Nikonenko *

Kuban State University, 149, Stavropolskaya Str., Krasnodar 350040, Russia
* Correspondence: v_nikonenko@mail.ru; Tel.: +7-918-41-45-816

Supplementary materials

S1 Solutions

Monosodium phosphate (NaH_2PO_4) is a salt of the tribasic orthophosphoric acid. The latter has the following structure

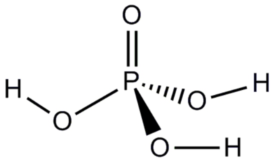

The proton-transfer reactions between water molecules and polybasic acid species (the cases of phosphoric with general formula H_nA, where the maximum value of n is 3) are presented as follows:

$$\mathrm{H_2A^- + H_2O \underset{k_{-1}}{\overset{k_1}{\rightleftarrows}} H_3A + OH^-}; \qquad K_{b1} = \frac{c_{H_3A} c_{OH^-}}{c_{H_2A^-}} \tag{S1}$$

$$\mathrm{H_3A + H_2O \underset{k_{-2}}{\overset{k_2}{\rightleftarrows}} H_2A^- + H_3O^+}; \qquad K_{a1} = \frac{c_{H_2A^-} c_{H^+}}{c_{H_3A}} \tag{S2}$$

$$\mathrm{HA^{2-} + H_2O \underset{k_{-3}}{\overset{k_3}{\rightleftarrows}} H_2A^- + OH^-}; \qquad K_{b2} = \frac{c_{H_2A^-} c_{OH^-}}{c_{HA^{2-}}} \tag{S3}$$

$$\mathrm{H_2A^- + H_2O \underset{k_{-4}}{\overset{k_4}{\rightleftarrows}} HA^{2-} + H_3O^+}; \qquad K_{a2} = \frac{c_{HA^{2-}} c_{H^+}}{c_{H_2A^-}} \tag{S4}$$

$$\mathrm{A^{3-} + H_2O \underset{k_{-5}}{\overset{k_5}{\rightleftarrows}} HA^{2-} + OH^-}; \qquad K_{b3} = \frac{c_{HA^{2-}} c_{OH^-}}{c_{A^{3-}}} \tag{S5}$$

$$\mathrm{HA^{2-} + H_2O \underset{k_{-6}}{\overset{k_6}{\rightleftarrows}} A^{3-} + H_3O^+}; \qquad K_{a3} = \frac{c_{A^{3-}} c_{H^+}}{c_{HA^{2-}}} \tag{S6}$$

Calculation of the mole fraction of ampholyte species in solution and membrane

Fig. S1 shows the distribution of species of the polybasic acids under study (in mole fractions) vs. the pH of the solution. These distributions are calculated using the appropriate equilibrium equations and the pK_a values presented in Table S1.

Table S1

The values of pK_a (at 25 °C) of acids [1], various species of which may be present in the membrane systems under study.

Substance	pK_{a1}	pK_{a2}	pK_{a3}
H_3PO_4	2.12	7.21	12.34

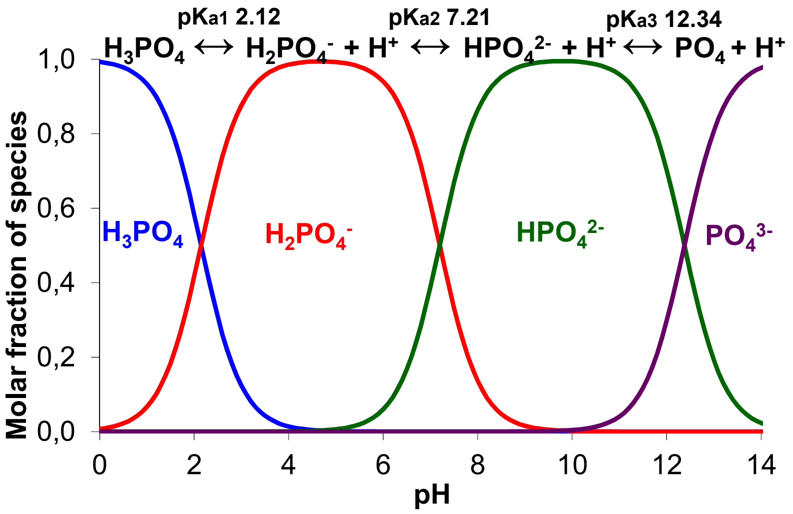

Fig. S1. Speciation diagrams: distribution of the phosphoric acid species (in mole fractions) vs. the pH of the solution.

S2. The Lévêque limiting current density and diffusion layer thickness.

S2.1. The case of a binary electrolyte (NaH_2PO_4, pH 4.6±0.1)

The Leveque equation [(S7) and equation (S8)] are used to estimate the theoretical limiting current, i_{lim}^{Lev}, and the thickness of the depleted diffusion layer, δ^{Lev}:

$$i_{\lim}^{Lev} = \frac{z_1 F D c_1^0}{h(T_1 - t_1)}\left[1.47\left(\frac{h^2 V_0}{LD}\right)^{1/3} - 0.2\right] \quad \text{(S7)}$$

$$\delta^{Lev} = 0.71 h\left(\frac{LD}{h^2 V_0}\right)^{1/3} \quad \text{(S8)}$$

Here z_1 is the charge number of counterion 1, F is Faraday constant, D и t_1 are the diffusion coefficient of the electrolyte and the electromigration transfer number of the counterion at infinite dilution of the solution. The transport number of counterions in the membrane, T_1, was considered

equal to one; c_1 is its molar concentration in the feed solution entering the DC, V_0 is the average linear velocity of the solution flowing between the membranes forming the DC, h is the distance between the membranes, L is the length of the channel. These equations were obtained for the diffusion-convection heat transfer [2] and after were adapted to diffusion-convection mass transfer in electrode [3] and ion exchange membrane [4] systems. For 1: 1 electrolyte and laminar hydrodynamic regime. Note that the value 0.71 for the factor in the right-hand part of Eq. (S8) is given by the numerical solution of the 2D convection-diffusion problem whose asymptotic solution is expressed by Eq. (S7). However, often [5,6] Eq. (S8) is used with a factor 0.68, which is obtained using the Peers equation [6] and the approximation of Eq. (S7), where the second term in the brackets is neglected. It is worth noting that this equation is applicable only for relatively short channel lengths ($L \le 0.02h^2V_0/D$) [4].

S2.2. The case of a mixed electrolyte solution

Let us consider a ternary electrolyte composed of two kinds of counterions, 1 and 2, and one kind of coion, a later on, we consider a solution of this ternary electrolyte in a diffusion layer adjacent to an ion-exchange membrane.

The Nernst-Planck equations for these ions read:

$$j_1 = -D_1\left(\frac{dc_1}{dx} + z_1 c_1 \frac{F}{RT}\frac{d\varphi}{dx}\right) \tag{S8}$$

$$j_2 = -D_2\left(\frac{dc_2}{dx} + z_2 c_2 \frac{F}{RT}\frac{d\varphi}{dx}\right) \tag{S9}$$

$$j_A = -D_A\left(\frac{dc_A}{dx} + z_A c_A \frac{F}{RT}\frac{d\varphi}{dx}\right) \tag{S10}$$

After dividing each of the equations (S8)-(S9) by D_i, summing the results, and taking into account the electroneutrality condition

$$z_1c_1 + z_2c_2 + z_Ac_A = 0 \tag{S11}$$

we find:

$$\frac{j_1}{D_1} + \frac{j_2}{D_2} + \frac{j_A}{D_A} = -\frac{d\left(c_1 + c_2 + c_3\right)}{dx} = -\frac{\left(1 + \left|z_1 / z_A\right|\right)dc_1}{dx} - \frac{\left(1 + \left|z_2 / z_A\right|\right)dc_2}{dx} \tag{S12}$$

The last equality in Eq. (S12) is obtained after eliminating c_A using Eq. (S11). Since j_i do not change along the coordinate x in a stationary state, Eq. (S12) can be easily integrated over the thickness of the diffusion layer. If the current density is equal to the limiting one, the concentrations of all ions at the membrane surface are very close to zero. In this case, we can write:

$$\frac{j_{1\lim}^{theor}}{D_1}+\frac{j_{2\lim}^{theor}}{D_2}=\frac{\left(1+\left|z_1/z_A\right|\right)c_1^0}{\delta}+\frac{\left(1+\left|z_2/z_A\right|\right)c_2^0}{\delta}-\frac{j_{A\lim}^{theor}}{D_A} \tag{S13}$$

The limiting flux density of counterion *i* can be represented as [7]:

$$j_{i\lim}^{theor}=\frac{\left(1+\left|z_i/z_A\right|\right)D_ic_i^0}{\delta}-\frac{\left|z_i\right|D_ic_i^0}{\left|z_A\right|D_Ac_A^0}\cdot j_{A\lim}^{theor} \tag{S14}$$

The first term in Eq. (S14) shows what value the limiting flux density of counterion *i* would have if the membrane were impermeable to coions. The second term reflects the additional value of the counterion flux caused by the transfer of coions: when the coions appear in the depleted layer, they create an additional electric field that attracts counterions from the solution. This effect is called exaltation in the literature [7,8].

From Eq. (S14), it is easy to obtain an expression for the limiting current density:

$$i_{\lim}=z_1j_1+z_2j_2+z_Aj_A=i_{\lim}^0+\left(\frac{T_{A_{\lim}}}{t_A}\right)i_{\lim} \tag{S15}$$

where $t_A=\frac{D_Az_A^2c_A^0}{\sum_{i=1,2,A}D_iz_i^2c_i^0}$ is the coion transport number in the bulk solution, $T_{A_{\lim}}=\frac{z_Aj_{A\lim}^{theor}F}{i_{\lim}}$ is the coion effective transport number in the membrane at $i=i_{\lim}$,

$$i_{\lim}^0=\frac{\left[\left(1+\left|z_1/z_A\right|\right)D_1z_1c_1^0+\left(1+\left|z_2/z_A\right|\right)D_2z_2c_2^0\right]F}{\delta} \tag{S16}$$

is the limiting current density in the case of a membrane impermeable to coions. The term $\left(T_{A_{\lim}}/t_A\right)i_{\lim}$ in Eq. (S17) can be interpreted as the sum of the current carried by the coions $\left(T_A\cdot i_{\lim}\right)$, and the exaltation current of counterions. From Eq. (S17), we obtain:

$$i_{\lim}=\frac{i_{\lim}^0\cdot t_A}{t_A-T_A} \tag{S18}$$

The resulting Eq. (S18) generalizes the well-known Peers equation for a single electrolyte. Indeed, setting $c_2^0=0$ gives:

$$i_{\lim}=\frac{D\cdot z_1\cdot c_1^0\cdot F}{\left(T_1-t_1\right)\delta} \tag{S19}$$

where

$$D=\frac{D_1D_A\left(z_1+\left|z_A\right|\right)}{D_1z_1+D_A\left|z_A\right|} \tag{S20}$$

is the electrolyte diffusion coefficient.

For a mixture of two single electrolytes with a common coion (a ternary electrolyte), one can obtain equation (S21), which is similar to equation (S19) [9]:

$$i_{\lim} = \frac{D_{ter} \cdot |z_A| \cdot c_A^0 \cdot F}{(t_A - T_A)\delta} \quad \text{(S21)}$$

with D_{ter}, the effective diffusion coefficient of ternary electrolyte,

$$D_{ter} = \left[\left(1+\left|\frac{z_1}{z_A}\right|\right)D_1 N_1 + \left(1+\left|\frac{z_2}{z_A}\right|\right)D_2 N_2\right]\cdot t_A = \frac{\left[\left(z_1+|z_A|\right)D_1 z_1 c_1^0 + \left(z_2+|z_A|\right)D_2 z_2 c_2^0\right]D_A}{\sum_{i=1,2,A} D_i z_i^2 c_i^0} \quad \text{(S22)}$$

$N_i = \frac{z_i c_i^0}{z_A c_A^0}$ is the equivalent fraction of counterion i in the bulk solution. It is easy to see that in the case, where the concentration of counterion 2 is zero, $c_2^0 = 0$, Eq. (S22) is reduced to Eq. (S20).

For a single electrolyte, the Leveque equation (Eq. S7), allows calculating the limiting current density and diffusion layer thickness as functions of the (single) electrolyte diffusion coefficient, solution flow rate, distance between the membranes and membrane length. It can be assumed that this equation remains valid in the case of ternary electrolyte, if the value determined by Eq. (S22) is used as the electrolyte diffusion coefficient. Under this assumption, we can write

$$\delta^{Lev} = 0.68h\left(\frac{LD_{ter}}{h^2 V_0}\right)^{1/3} \quad \text{(S23)}$$

Table S2 summarizes some of the characteristics of the studied electrolytes, which are used to calculate the limiting currents.

Table S2

Some of the characteristics of the studied electrolytes, which are used to calculate the limiting currents.

Electrolyte	Diffusion coefficients at infinite dilution, $D_i \times 10^5$, cm^2 s^{-1}				Transport numbers at infinite dilution, t_i		
	cation	anion		electrolyte	cation	anion	
		singly charged	doubly charged			singly charged	doubly charged
NaCl	1.334 [1]	2.032 [1]		1.61	0.396	0.604	
NaH_2PO_4		0.959 [1]		1.12	0.581	0.419	
Na_2HPO_4		-	0.759 [1]				0.456

Calculations made using equation S22 give the following value $D_{ter} = 1.29\times10^5$, cm^2 s^{-1} It is calculated under the assumption that Na^+ is the only coion.

S3. ***The effective transport numbers***, T_i ***and partial current numbers*** i_i ***of counter ions***

The effective transport numbers, T_i *and partial current numbers* i_i *of counter ions* were found using the cell presented in Fig. 2 (the main text). The difference with the measurements of current voltage curves is in the fact that desalination compartment (14) is fed from additional tank (2). The volume of the solution circulating through the desalination compartment (DC) and tank (2) is 0.1 L, which is essentially less than the volume of the solution circulating through tank (1), the concentration and electrode compartments. During one experimental run, in conditions where the potential difference between the Luggin capillaries, $\Delta\varphi$, is kept constant, the salt concentration in the desalination circuit decreased with time. Since the rates of generation of H^+ and OH^- ions at the CEM and AEM forming the desalination compartment are different, pH of the feed solution changed with time. Namely, it became acidic in the studied cases. In order to keep a constant pH value of the feed solution, a 0.1M solution of NaOH was added into tank (2) through microcapillary (13). The rate of decrease in the salt concentration of the solution in tank (2), *dC/dt*, is found by using the measured values of conductivity, κ, of this solution (using submersible conductometric cell (12)) and taking into account the known constant pH value. The effective transport numbers, T_i, of salt and water ions in the anion-exchange membrane under study (AEM* in Fig. 2) are found by knowing the rate of concentration decrease *dC/dt*, and the rate of addition of NaOH into tank (2); the mass balance equations are applied in the calculations. Simultaneously, the partial current density of species "*i*", i_i, is determined as $i_i = i\, T_i$.

For the desalted *NaCl solution*, the material balance of the transport of counterions in the desalting channel is described by Eq. (S24), if we assume that the difference in electrolyte concentration in different parts of the installation (tube, cell, vessel) is insignificant:

$$\overline{V}\frac{dC}{dt} = -\frac{i(T_1^{CEM} - T_1^{AEM})S\,n}{z_1 F} + C_T\frac{d\overline{V_T}}{dt} \qquad \text{(S24)}$$

Here T_i^{CEM}, T_i^{AEM} are the effective numbers of counterions in the cation-exchange and anion-exchange membranes that form the desalting channel; S is the active (polarizable) surface area of the membrane under study; n is the number of desalination chambers; $\overline{V}$ is the volume of the solution in the desalting channel; C_T and $\overline{V_T}$ are concentration and volume of the titrant (NaOH) added in the desalination path. The first term in the right-hand side of Eq. (S24) describes the decrease in the concentration of the electrolyte in the DC due to the transfer of counterions through the corresponding membranes; the second term of the equation describes the addition of sodium ions with the titrant.

In the case when the transfer of salt co-ions through membranes can be neglected (since 0.02 M solutions used in the study are quite diluted), it follows from Eq. (S24):

$$j_{Na^+}^{CEM} = \frac{i_{Na^+}^{CEM}}{F} = \frac{iT_{Na^+}^{CEM}}{F} \approx -\frac{\overline{V}}{S}\frac{dC}{dt} + \frac{C_T}{S}\frac{d\overline{V_T}}{dt} \qquad \text{(S25)}$$

$$j_{Cl^-}^{AEM} = \frac{i_{Cl^-}^{AEM}}{F} = \frac{iT_{Cl^-}^{AEM}}{F} \approx -\frac{\overline{V}}{S}\frac{dC}{dt} \qquad \text{(S26)}$$

After the effective transfer numbers of salt counterions have been determined, it is easy to find the effective transfer numbers of protons (CEM) and hydroxyl ions (AEM):

$$T_{H^+}^{CEM} = 1 - T_{Na^+}^{CEM}, \qquad T_{OH^-}^{AEM} = 1 - T_{Cl^-}^{AEM} \qquad \text{(S27)}$$

In the case of desalting a *NaH_2PO_4 solution*, the partial currents of protons and sodium ions in the CEM are calculated using Eqs. (S26) and (S27).

To determine the partial currents of $H_2PO_4^-$, HPO_4^{2-}, PO_4^{3-} and OH^- ions in an AEM, the material balance equations were composed on the basis of the following assumptions.

1. The total flux of pentavalent phosphorus (P) in the AEM is equal to the sum of all fluxes of phosphorus-containing ions entering the AEM from the desalination channel. Since pH in the intermediate tank, from which the solution enters the DC, is 4.6, only $H_2PO_4^-$ ions are present in it (Fig. S1). In this case:

$$j_{H_2PO_4^-}^{s} = j_P^{AEM} = j_{H_2PO_4^-}^{AEM} + j_{HPO_4^{2-}}^{AEM} + j_{PO_4^{3-}}^{AEM} = \frac{i_{H_2PO_4^-}^{AEM}}{z_{H_2PO_4^-}F} + \frac{i_{HPO_4^{2-}}^{AEM}}{z_{HPO_4^{2-}}F} + \frac{i_{PO_4^{3-}}^{AEM}}{z_{PO_4^{3-}}F} \qquad \text{(S28)}$$

where the "AEM" and "s" indices refer to the anion-exchange membrane and the solution on its side adjacent to the depleted diffusion layer (Fig. 6 in the main text), respectively. The value of $j_{H_2PO_4^-}^{s}$ can be easily determined experimentally from the rate of concentration decrease in the desalting stream (where the pH value is kept constant) using an equation similar to Eq. (S26):

$$j_{H_2PO_4^-}^{s} = -\frac{\overline{V}}{S}\frac{dC_{NaH_2PO_4}}{dt} \qquad \text{(S29)}$$

It follows from Fig. S1 that only two types of phosphorus-containing particles can be simultaneously present in the AEM, namely $H_2PO_4^-$ and HPO_4^{2-} or HPO_4^{2-} and PO_4^{3-}. The concentration of other particles in each of the three pH ranges is very low and they can be neglected. A third pair of PO_4^{3-} and OH^- ions can coexist at pH > 13.

It should be noted that protons are co-ions and are excluded from the AEM due to the Donnan effect [10]. Therefore, the pH of the internal membrane solution is 1–2 units higher than the pH of the external solution [11]. It follows from the Donnan equation that the electrostatic exclusion of co-ions increases with dilution of the external solution [11]. The results of

mathematical simulation, which take into account the values of the protonation–deprotonation constants of orthophosphoric acid particles (Table S1) and the Donnan potential of the AEM [11], show that as the current density in the membrane system increases, the solution in contact with the surface of the AEM from the depleted diffusion layer becomes more and more diluted. In this case, the concentration of H^+ ions in the near-surface layer of the membrane decreases, and the pH of this layer increases. At a relatively low current density (low concentration polarization), the pH of the near-surface AEM layer is relatively low, and this layer contains $H_2PO_4^-$ and HPO_4^{2-} ions. With an increase in the current density, the pH of the near-surface layer of the AEM increases; it is enriched first with PO_4^{3-} and then with OH^-.

In the pH range from 5 to 10 (relatively low current densities), when there are only $H_2PO_4^-$ and HPO_4^{2-} ions in the AEM, the total current density, *i*, is determined by the transfer of only these ions:

$$i_{H_2PO_4^-}^{AEM} + i_{HPO_4^{2-}}^{AEM} = i \quad \text{(S30)}$$

and, in accordance with Eq. (S29),

$$j_{H_2PO_4^-}^{s} = \frac{i_{H_2PO_4^-}^{AEM}}{F} + \frac{i_{HPO_4^{2-}}^{AEM}}{2F} \quad \text{(S31)}$$

In this case, the partial currents of singly and doubly charged phosphorus-containing ions can be found by the following equations:

$$i_{H_2PO_4^-}^{AEM} = 2Fj_{H_2PO_4^-}^{s} - i \quad \text{(S32)}$$

$$i_{HPO_4^{2-}}^{AEM} = 2\left(i - Fj_{H_2PO_4^-}^{s}\right) \quad \text{(S33)}$$

In the pH range from 10 to 13.5 (high current densities), we find in a similar way:

$$i_{HPO_4^{2-}}^{AEM} = 2\left(3Fj_{H_2PO_4^-}^{s} - i\right) \quad \text{(S34)}$$

$$i_{PO_4^{3-}}^{AEM} = 3\left(i - 2Fj_{H_2PO_4^-}^{s}\right) \quad \text{(S35)}$$

Coefficients «2» and «3» in Eqs. (S32) – (S35) correspond to electric charges $z_{HPO_4^{2-}}$ and $z_{PO_4^{3-}}$.

At higher current densities, when doubly charged ions are transformed into triply charged ones ($T_{HPO_4^{2-}} = i_{HPO_4^{2-}} / i = 0$), and the pH of the surface layer of the AEM exceeds 13.5, the current in the membrane is determined by the transfer of PO_4^{3-} and OH^-. The partial currents of these ions are found as:

$$i_{PO_4^{3-}}^{AEM} = 3Fj_{H_2PO_4^-}^{s} \quad \text{(S36)}$$

$$i_{OH^-}^{AEM} = i - i_{PO_4^{3-}}^{AEM} \quad (S37)$$

The partial flux of protons entering the depleted diffusion layer (DDL) is:

$$j_{H^+}^{s} = j_{HPO_4^{2-}}^{AEM} + 2j_{PO^{3-}}^{AEM} \quad (S38)$$

Taking into account that $i_k = j_k z_k F$, the partial current density of H^+ ions in the depleted diffusion layer at the membrane surface is:

$$i_{H^+}^{s} = \frac{i_{HPO_4^{2-}}^{AEM}}{2} + \frac{2i_{PO_4^{3-}}^{AEM}}{3} \quad (S39)$$

Note that the i^S_{H+} values are not difficult to find if the concentration (C_T) and flow rate of the titrant (W_T), continuously added to the vessel of the experimental setup are known (Fig. 6 in the main text). In this case, the equation for calculating i^S_{H+} is:

$$i_{H^+}^{s} = -\frac{FW_T}{S}\frac{dC_T}{dt} \quad (S40)$$

Eq. (S40) is derived under the assumption that all protons entering the solution from the AEM/solution interface are carried out by the fluid flow from the desalination channel. This equation is convenient to use, if the solution entering the desalting channel simultaneously contains not one, but several anions (solutions of tartaric and citric acid salts), and the calculation of the partial fluxes of these anions in the membrane is difficult. The confidence interval for determining the effective transport number of counterion in membrane for a given current density is equal to ± 0.03.

References

1. Lide, D.R. *CRC Handbook of Chemistry and Physics 86TH Edition 2005-2006*; 2005; ISBN 0849304792.
2. Lévêque M. A. The laws of heat transmission by convection. *Les Ann. des Mines Mem.* **1928**, *12*, 201–299.
3. Newman J., T.-A.K.E. *Electrochemical systems*; John Wiley & Sons, 2012;
4. N.P. Gnusin, V.I. Zabolotskii, V.V. Nikonenko, M.K.U. Convective-Diffusion Model of Electrodialytic Desalination. Limiting Current and Diffusion Layer. *Sov. Electrochem.* **1986**, *23*, 273–278.
5. La Cerva, M.; Gurreri, L.; Tedesco, M.; Cipollina, A.; Ciofalo, M.; Tamburini, A.; Micale, G. Determination of limiting current density and current efficiency in electrodialysis units. *Desalination* **2018**, *445*, 138–148, doi:10.1016/j.desal.2018.07.028.
6. Nikonenko, V.; Nebavsky, A.; Mareev, S.; Kovalenko, A.; Urtenov, M.; Pourcelly, G. Modelling of Ion Transport in Electromembrane Systems: Impacts of Membrane Bulk and

Surface Heterogeneity. *Appl. Sci.* **2018**, *9*, 25, doi:10.3390/app9010025.

7. Urtenov, M.A.K.; Kirillova, E. V.; Seidova, N.M.; Nikonenko, V. V. Decoupling of the Nernst-Planck and Poisson equations. Application to a membrane system at overlimiting currents. *J. Phys. Chem. B* **2007**, doi:10.1021/jp073103d.
8. Kharkats, Y.I.; Sokirko, A.V. Theory of the effect of migration current exaltation taking into account dissociation-recombination reactions. *J. Electroanal. Chem.* **1991**, *303*, 27–44, doi:10.1016/0022-0728(91)85113-4.
9. Titorova, V.D.; Mareev, S.A.; Gorobchenko, A.D.; Gil, V.V.; Nikonenko, V.V.; Sabbatovskii, K.G.; Pismenskaya, N.D. Effect of current-induced coion transfer on the shape of chronopotentiograms of cation-exchange membranes. *J. Memb. Sci.* **2021**, *624*, 119036, doi:10.1016/j.memsci.2020.119036.
10. Helfferich, F.G. Ion-exchange membrane separation processes. *Sep. Purif. Technol.* **2005**, *42*, 101, doi:10.1016/j.seppur.2004.05.007.
11. Sarapulova, V.; Nevakshenova, E.; Pismenskaya, N.; Dammak, L.; Nikonenko, V. Unusual concentration dependence of ion-exchange membrane conductivity in ampholyte-containing solutions: Effect of ampholyte nature. *J. Memb. Sci.* **2015**, *479*, 28–38, doi:10.1016/j.memsci.2015.01.015.
